# Claudin-6 and Occludin Natural Variants Found in a Patient Highly Exposed but Not Infected with Hepatitis C Virus (HCV) Do Not Confer HCV Resistance *In Vitro*

**DOI:** 10.1371/journal.pone.0142539

**Published:** 2015-11-12

**Authors:** Lucie Fénéant, Jade Ghosn, Baptiste Fouquet, François Helle, Sandrine Belouzard, Thibaut Vausselin, Karin Séron, Jean-François Delfraissy, Jean Dubuisson, Micheline Misrahi, Laurence Cocquerel

**Affiliations:** 1 Univ. Lille, CNRS, Inserm, CHU Lille, Institut Pasteur de Lille, U1019—UMR 8204—CIIL—Centre d'Infection et d'Immunité de Lille, F-59000 Lille, France; 2 Assistance Publique—Hôpitaux de Paris, Unité Fonctionnelle de Thérapeutique en Immuno-Infectiologie, Hôpital Universitaire Hôtel Dieu, Paris, France; 3 Université Paris Descartes, EA 7327, Faculté de Médecine site Necker, Paris, France; 4 Univ Paris Sud, Faculté de Médecine, Hôpitaux Universitaires Paris Sud, Le Kremlin-Bicêtre and Inserm-U1193, Hôpital Paul Brousse, F-94800 Villejuif, France; 5 Virology Department, Amiens University Hospital, Amiens, France; 6 Assistance Publique—Hôpitaux de Paris, Service de Médecine Interne et Maladies Infectieuses, Centre Hospitalier Universitaire de Bicêtre, Le Kremlin-Bicêtre, France; SAINT LOUIS UNIVERSITY, UNITED STATES

## Abstract

The clinical course of Hepatitis C Virus (HCV) infection is highly variable between infected individual hosts: up to 80% of acutely HCV infected patients develop a chronic infection while 20% clear infection spontaneously. Spontaneous clearance of HCV infection can be predicted by several factors, including symptomatic acute infection, favorable *IFNL3* polymorphisms and gender. In our study, we explored the possibility that variants in HCV cell entry factors might be involved in resistance to HCV infection. In a same case patient highly exposed but not infected by HCV, we previously identified one mutation in claudin-6 (CLDN6) and a rare variant in occludin (OCLN), two tight junction proteins involved in HCV entry into hepatocytes. Here, we conducted an extensive functional study to characterize the ability of these two natural variants to prevent HCV entry. We used lentiviral vectors to express Wildtype or mutated CLDN6 and OCLN in different cell lines and primary human hepatocytes. HCV infection was then investigated using cell culture produced HCV particles (HCVcc) as well as HCV pseudoparticles (HCVpp) expressing envelope proteins from different genotypes. Our results show that variants of CLDN6 and OCLN expressed separately or in combination did not affect HCV infection nor cell-to-cell transmission. Hence, our study highlights the complexity of HCV resistance mechanisms supporting the fact that this process probably not primarily involves HCV entry factors and that other unknown host factors may be implicated.

## Introduction

Hepatitis C is a global health problem with more than 160 million infected people worldwide [1]. An estimated additional two million people are newly infected per year, most of them through contaminated needle injections [2]. Hepatitis C Virus (HCV) prevalence is estimated to 1.8% in the USA and goes up to 75% for intravenous drug users patients (IVDU) [3]. As HCV and HIV (Human Immunodeficiency Virus) share the same transmission routes, they are frequently found concomitantly, in particular for highly exposed populations such as IVDU. For patients infected with HIV, the seroprevalence for HCV is around 24% [4]. However, this seroprevalence can be very different, depending on the population studied. Indeed, this seroprevalence is less than 10% for homo- and bi-sexuals patients, 41.7% for haemophiliac and transfusion recipients, while it can reach 92.8% for IVDU [3]. As a consequence, it is considered as a relatively rare event for IVDU not to be infected by HCV when they have already contracted HIV, as these patients are highly exposed to HCV.

HCV is a small enveloped positive single stranded RNA virus, belonging to the Hepacivirus genus in the *Flaviviridae* family. Its genome encodes an approximately 3000 amino acid polyprotein which is maturated into structural, E1 and E2 glycoproteins and the capsid protein core, and non structural proteins [5]. E1 and E2 envelope glycoproteins are known to play a key role in HCV entry into hepatocytes, the major target of HCV, by interacting with a series of cellulars factors. Indeed, HCV entry is a complex multistep process requiring many specific entry factors. HCV infection begins with the attachment of the viral particle to the cell surface of hepatocytes through attachment factors such as glycosaminoglycans and low density lipoproteins receptor [6,7]. This attachment allows the contact between the viral particle and specific cell entry factors, including the tetraspanin CD81 [8], the scavenger receptor class B type 1 (SRB1) [9] and the tight junction proteins claudin-1 (CLDN1) [10] and occludin (OCLN) [11,12]. Interestingly, two other tight junction proteins, CLDN6 and CLDN9, were described as cofactors that HCV is able to use instead of CLDN1 in certain cell types [13,14]. Additional entry factors have been described more recently such as tyrosine kinase epidermal growth factor-receptor (EGF-R) and Ephrin A2 receptor [15], the Niemann-Pick C1-like 1 receptor [16], the transferrin receptor [17] and the tetraspanin CD63 [18]. However, their exact role in HCV entry still needs to be investigated. After interacting with these factors, HCV particles are internalized through a clathrin-mediated endocytosis [19,20] and the viral RNA is released into the cytosol through the fusion of the viral envelope at low pH with the membrane of an early endosome [21,22].

In a previous study, we recruited a cohort of IVDU patients infected by HIV highly exposed but not infected with HCV [23] and we sequenced major HCV entry factors for these patients [24]. In one patient, we found two heterozygous variants, one mutation in CLDN6 not found in databases and one rare variant in OCLN. These mutations affect residues that are highly conserved in different species and were predicted to be damaging [24]. These mutations were not detected in the control population of IVDU HIV+ HCV+ patients. Therefore, in the present study, we hypothesized that these mutations could be related to the HCV resistance of the patient. We extensively characterized the functionality of these mutations in HCV entry and cell-to-cell spread; we used different cellular models and HCV genotypes to analyze their effect on HCV infection.

## Materials and Methods

### Cell culture

Dulbecco’s modified Eagle’s medium (DMEM), William’s medium E, Opti-MEM, phosphate-buffered saline (PBS), Glutamax-I, non-essential amino acids (NEAA), goat and fetal bovine serum (FBS) were from Invitrogen. DMSO and Poly-D-lysine hydrobromide were purchased from Sigma-Aldrich. 4’6-Diamidino-2-phenylindole (DAPI) was purchased from Molecular Probes (Invitrogen). Mowiol 3–88 is from Calbiochem. Huh-7 and 293T cells were from ATCC. TZM cells were from the NIH AIDS reagent Program. Huh-7-Lunet-CD81-Fluc-BLR cells [25] were kindly provided by T. Pietschmann (Twincore, Hanover, Germany). 786-O cells were kindly provided by Mariana Varna (Hôpital St Louis, Paris). HepG2-CD81-1SC3 clone was isolated from HepG2 cells expressing CD81 and selected for their capacity to polarize like simple epithelial cells when grown on semi-permeable support (Transwells, Costar) in William’s medium E supplemented with 10% FBS and 1% DMSO (S. Belouzard, unpublished data). The polarization of the cells was confirmed by the localization of apical, basolateral and tight junctions markers and by the secretion of human serum albumin in the basolateral chamber.

### Antibodies

Mouse anti-E1 (A4) [26] and anti-E2 (3–11, kindly provided by J. McKeating, University of Birmingham, United Kingdom) [27] antibodies were produced *in vitro* by using a MiniPerm apparatus (Heraeus), as recommended by the manufacturer. The anti-NS5 (2F6/G11) was from Austral Biologicals. The anti-CLDN6 antibody (clone 342927, Antibody Registry # AB_2292076, targeting an extracellular epitope) was from R&D Systems. Secondary antibodies were from Jackson Immunoresearch.

### Lentivectors cloning and production

Wildtype (wt) and mutant CLDN6 (R209Q) were cloned into pTRIP vector kindly provided by C. M. Rice (The Rockefeller University, New York, USA). Since there is no antibody directed against the extracellular domains of OCLN, we fused OCLN to GFP, at its C-terminus to avoid any effect of the GFP on the N-terminal mutation. GFP-tagged wt and mutant OCLN (P24A) were also cloned into pTRIP vector. pTRIP-YFP, pTRIP-SRB1, pTRIP-venus-OCLN, pTRIP-cerulean-CLDN1 and pTRIP-mcherry-CD81 were described previously [11] and kindly provided by C. M. Rice. Lentivectors were produced by transfecting 293T cells with pTRIP constructs, phCMV-VSVG and HIV gag-pol, with Exgen 500 (Euromedex) according to the manufacturer instructions. Medium was changed 6 h after transfection and cells were incubated at 32°C for 72 h. Supernatants containing lentivectors were filtered at 0.45 μm.

### Transductions

Supernatants containing lentivectors were added to 293T, 786-O, TZM, Huh-7, Lunet-CD81, or HepG2-CD81-1SC3 cells seeded the day before in 24-well plates to a ratio of 1:4 in serum free DMEM. For infection of PHH, a ratio of 1:2 was used. Cells were incubated for 4 h at 37°C and DMEM 10% FBS was added. Cells were transduced twice at 24 h interval. When transductions with multiple lentivectors was required, cells were transduced sequentially twice with each lentivectors and were split (not for PHH) between each transduction with a different lentivector. 293T cells were seeded on poly-D-Lysine coated plates for transductions.

### HCVpp production and infection

HCVpp and VSVpp were produced as described previously [28,29] with plasmids kindly provided by B. Bartosch and F.L. Cosset (Lyon, France). Pseudotyped particles were inoculated on 293T, 786-O or Huh-7 cells for 3 h at 37°C. At 72 h post-infection, cells were lysed and processed to measure the *Firefly* luciferase activities as indicated by the manufacturer (Promega). Luciferase activities with HCVpp were normalized for luciferase activities obtained with VSVpp. In each figure, results are reported as the mean ± S.D. of at least three independent experiments.

### HCVcc and infection assays

HCVcc used in this study were based on the JFH1 strain [30] and contained cell culture-adaptive mutations [31,32]. The JFH1-based intergenotypic chimeras were kindly provided by J. Bukh (University of Copenhagen, Denmark) [33]. HCVcc (JFH1/CSN6A4/5’C19Gluc2Aubi) expressing the *Gaussia*-luciferase reporter gene were produced as previously described [31,34].

HCVcc were added to cells (m.o.i. = 1) seeded the day before in 24-well plates and incubated for 2 h at 37°C. The supernatants were then removed and the cells were incubated in DMEM 10% FBS at 37°C. At 30 h post-infection, *Gaussia*-luciferase assays were performed as indicated by the manufacturer (Promega). Infections with JFH1-based intergenotypic chimeras were performed as described above. Infection levels were quantified by indirect immunofluorescence. In each figure, results are reported as the mean ± S.D. of at least three independent experiments.

### Indirect immunofluorescence microscopy

Transduced cells grown on coverslips were fixed with formalin solution (Sigma-Aldrich) or methanol. Cells were then stained with anti-CLDN6 mAb followed by CY3-conjugated goat anti-mouse and DAPI staining. Coverslips were mounted on glass slides using Mowiol, and observed with a Zeiss Axioplan 2 Axiophot 2 equipped with a 40×/1.3 numerical aperture lens.

Infected cells grown in 96-well plates were processed for immunofluorescence detection of E1 envelope protein with A4 mAb, as previously described [34]. Cells were observed with a Zeiss Axiophot equipped with a 10x magnification, 0.5 numerical aperture objective. Fluorescent signals were collected with a Coolsnap ES camera (Photometrix) using specific fluorescence excitation and emission filters. Images were processed with Image J software. For quantification of infection levels, images of randomly picked areas from each well were recorded and processed using ImageJ software. Cells labeled with anti-E1 mAb A4 were counted as infected cells. The total number of cells was obtained from DAPI-labeled nuclei. The infections were scored as the ratio of infected cells to total cells.

### Flow cytometry

Cells grown in 24-well plates were directly washed with PBS 2% BSA. Cells were then incubated for 1 h at 4°C with anti-CLDN6 mAb. After rinsing, cells were incubated with PE-labelled goat anti-mouse for 45 min at 4°C, washed, detached with PBS 2 mM EDTA and fixed with formalin solution. For OCLN expression, cells were washed, detached with PBS 2 mM EDTA and fixed with formalin solution. Cells were next analyzed with a FACSCalibur (Becton Dickinson).

### HCVcc cell-to-cell transmission assay

Twenty-four hours prior to their use in the assay, Huh-7 cells were infected with HCVcc expressing structural proteins from genotype 2a (HCVcc2a) or genotype 3a (HCVcc3a) (m.o.i = 2). Infected cells were then labeled with 5 chloromethylfluorescein diacetate (CMFDA) (Molecular Probes, Invitrogen) by incubation for 30 min at 37°C with 10 μM CMFDA in medium without FBS. Cells were trypsinized, washed and incubated for 15 min with 3–11 mAb (50 mg/ml) to neutralize any remaining viral particles at the cell surface. After washing, CMFDA-labeled donor cells were mixed with naïve target cell lines (ratio 1:4), seeded in 24-well plates in the presence (cell-to-cell transmission) or in the absence (cell-free and cell-to-cell transmission) of neutralizing mAb (3–11, 50 μg/ml). After 24 h, *de novo* transmission events were determined by staining for HCV NS5 and were quantified by flow cytometry, as described above. Cell-to-cell transmission levels were defined as newly infected cells in the presence of 3–11 mAb. Cell-free transmission levels were evaluated using the following calculation: (*de novo* infected cells in the absence of 3–11)—(*de novo* infected cells in the presence of 3–11).

### HepG2-CD81-1SC3 polarization and infection

HepG2-CD81-1SC3 cells were seeded the day before transduction on 6-well plates coated with gelatin in DMEM 10% FBS. Cells were transduced twice at 24 h interval. 24 h after the last transduction round, cells were plated on transwells in DMEM 10% FBS until confluence was reached. Next, medium was replaced by William’s Medium E 1% DMSO. Polarization was verified by anti-ZO-1 mAb staining, analyzed by indirect immunofluorescence microscopy (data not shown). Cells were infected with HCVcc2a by 1 h incubation of the virus on basolateral chamber membrane of the transwell. Supernatants and cell lysates were collected 30 h after infection and RNA were extracted with NucleoSpin RNA II kit (Macherey-Nagel) according to the manufacturer instructions. HCV RNA was quantified by quantitative real-time RT-PCR assay as described previously [35].

### Primary human hepatocytes infection assays

Primary human hepatocytes (PHH) were from Biopredic. They were infected with cell culture adapted HCVcc (JFH1-i24), as previously described [36]. Virus that had been inactivated at 60°C for 30 min was used as a control. Infection levels were evaluated by quantifying HCV RNAs, as described in [36].

## Results

### Description of CLDN6 and OCLN variants found in a patient highly exposed but not infected by HCV

Claudin proteins are important structural and functional components of tight junctions in paracellular transport. Complexed with two other integral transmembrane proteins, occludin and junctional adhesion molecule, claudins are located in both epithelial and endothelial cells in all tight junction bearing tissues. In hepatocytes, tight junction proteins are critical in maintaining the polarity.

**Fig 1:**
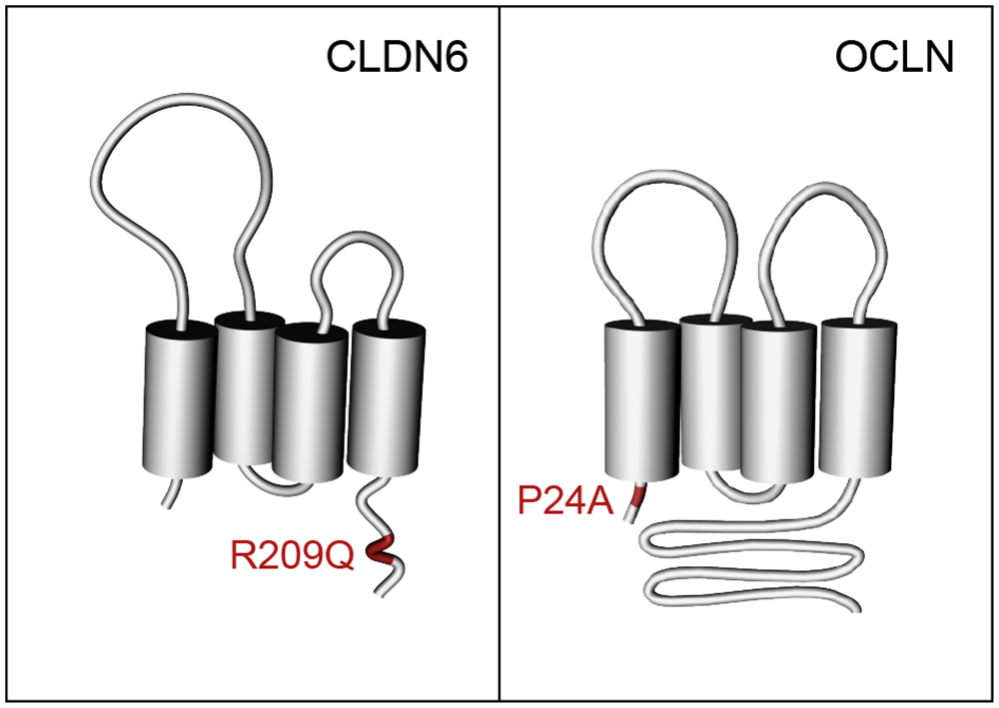
Schematic representation of mutated entry factors. Schematic representation of CLDN6 and OCLN tight junction proteins and position of R209Q and P24A mutations in CLDN6 and OCLN, respectively.

CLDNs and OCLN proteins consist of four transmembrane domains, two extracellular loops and two cytosolic domains (Fig 1). CLDN1 and OCLN have been identified as two essential entry factors for HCV [10,11]. CLDN1 is able to render 293T cells permissive for HCV entry and antibodies directed against CLDN1 inhibit *in vitro* and *in vivo* infection [10,37–39]. Two other members of the CLDN family, CLDN6 and CLDN9, are also able to render 293T cells permissive for HCV entry [13,14]. The first extracellular loop of CLDN1, through specific residues, is involved in HCV entry [10,40]. CLDN1 and the tetraspanin CD81 associate to form co-receptor complexes that are critical for HCV entry [37,41,42].

OCLN is also an essential HCV entry factor [11]. Its exact role is poorly understood but it is probably involved in a late post-binding step of entry and it is important to HCV species tropism. The second part of the second loop is required for HCV entry [11,43–45].

In a previous study, we conducted a case-control study in which we sequenced five entry factor gene coding regions in HIV-infected IVDU at high risk of HCV infection but not infected (cases) and HIV/HCV-coinfected IVDU (controls) [23,24]. Cases were defined as: (i) having acquired HIV-1 infection before 1995 through intravenous drug use, (ii) having injected illicit drugs for at least 5 years, and (iii) being negative for anti-HCV antibodies and HCV RNA. Controls were defined as: (i) having acquired HIV-1 infection before 1995 through intravenous drug use, (ii) having injected illicit drugs for at least 5 years, and (iii) being positive for anti-HCV antibodies and HCV-RNA before any anti-HCV treatment.

In a same HIV+ HCV- IVDU patient, we identified single nucleotide polymorphisms (SNPs) in *CLDN6* and *OCLN* genes: one SNP (rs149605777) corresponding to a c.626G>A heterozygous coding variant leading to a missense Arg209Gln (R209Q) change in the C-terminal cytosolic tail of CLDN6 (Fig 1), with non attributed MAF (minor allele frequency) in 1000 Genomes database and one rare heterozygous variant (rs147125035) corresponding to a missense c.70C>G substitution with a MAF frequency of 0.006. This latter mutation leads to a Pro24Ala (P24A) change in the N-terminus of OCLN (Fig 1). It has to be noted that both residues mutated in CLDN6 and OCLN are highly conserved in different species and prediction tools (PolyPhen 2 and SIFT) showed that the CLDN6 mutation is possibly damaging whereas the OCLN variant is predicted to be potentially damaging or deleterious. Strikingly, both variants were found in the same case patient that has been highly exposed but not infected by HCV [24]. Therefore, we hypothesized that these two natural variants may instigate HCV resistance of the patient.

### Expression and functionality in HCV entry of CLDN6/R209Q and OCLN/P24A expressed in 293T and 786-O cells

To characterize the functionality of CLDN6 and OCLN variants in HCV infection, we used HCVpp that are retroviral particles pseudotyped with HCV E1E2 envelope proteins from different genotypes (1a, 2a …) or control envelope protein (VSV-G) [28,46]. HCVpp enable to study specifically the entry step of the HCV lifecycle. We also used HCVcc that are infectious particles produced in cell culture and which allow for the study of the whole lifecycle of HCV [30,47,48].

**Fig 2:**
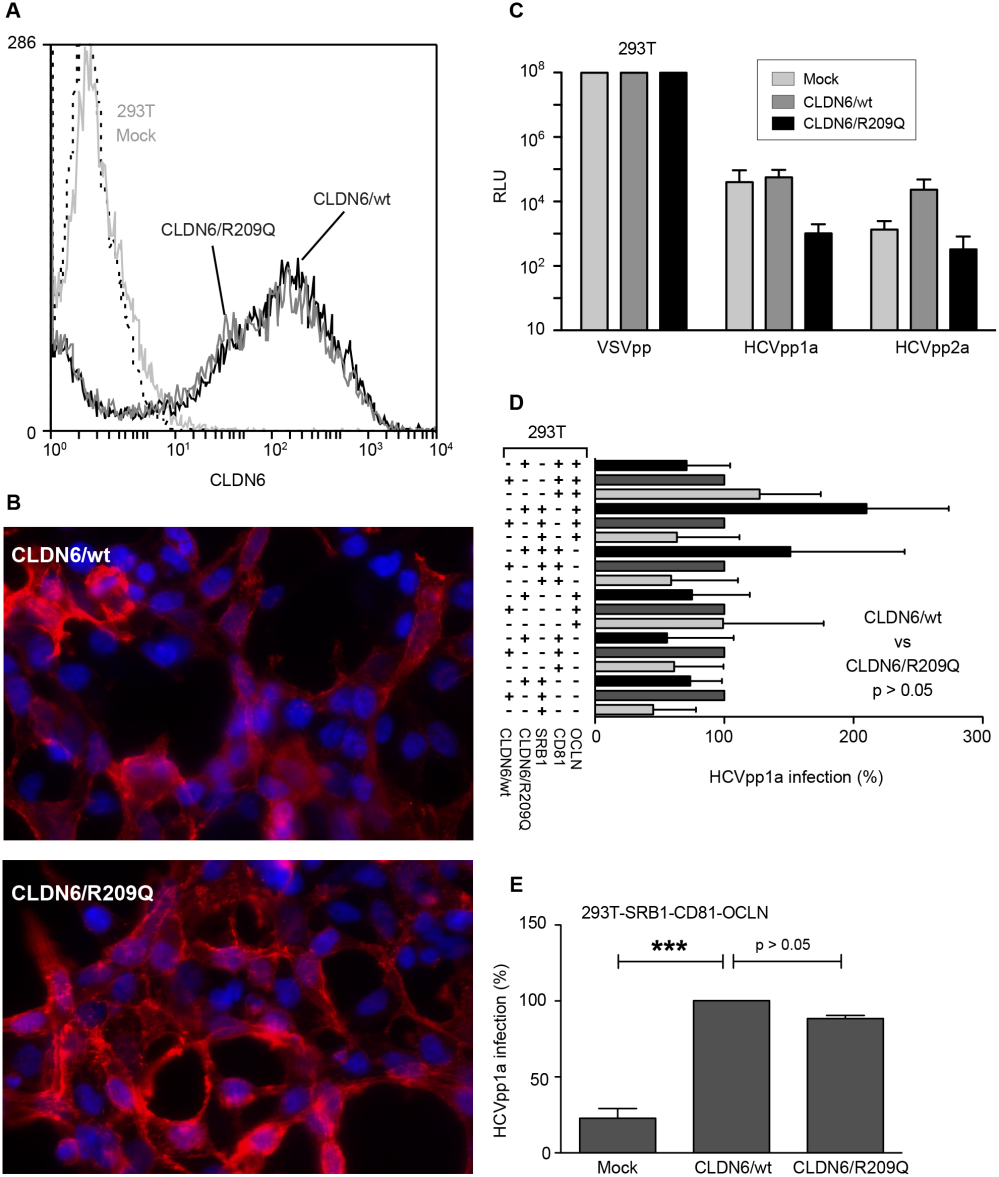
CLDN6/R209Q mutation does not affect HCVpp entry. 293T cells were transduced twice at 24h interval with CLDN6/wt or CLDN6/R209Q. Expression was analysed 48h after the last transduction round. (A) CLDN6/wt and CLDN6/R209Q cell surface expression was measured by flow cytometry using an anti-CLDN6 antibody, and compared to that of non-transduced 293T cells (293T Mock). Cells stained with secondary antibodies were used as negative controls (dashed line). (B) CLDN6/wt and CLDN6/R209Q expression in 293T cells was also analyzed by immunofluorescence with an anti-CLDN6 antibody. (**C**) Two days after the last transduction, 293T cells were infected with HCVpp1a, HCVpp2a or VSVpp expressing the *Firefly*-luciferase reporter gene. Non-transduced 293T cells (Mock) were infected in parallel. Results are presented in relative luciferase units (RLU). (D) 293T cells were transduced sequentially with a combination of lentivectors encoding SRB1, CD81 and/or OCLN in addition to CLDN6/wt or CLDN6/R209Q. Cells were next infected with HCVpp1a or VSVpp 48h after the last transduction round. Cells were lysed 72h post-infection and results were normalised with luciferase activities measured in cells infected with VSVpp. Values were adjusted to 100% infection for cells expressing CLDN6/wt. (**E**) 293T cells transduced to express SRB1, CD81 and OCLN in addition to CLDN6/wt or CLDN6/R209Q were infected with HCVpp1a or VSVpp. Cells were lysed 72h post-infection and results were normalised with luciferase activities measured in cells infected with VSVpp. Values were adjusted to 100% infection for cells expressing CLDN6/wt. Results are presented as mean ± SD of three independent experiments. *** means a *p* value below 0.001.

Since it has been described that 293T cells become permissive to HCVpp, when expressing CLDN6 or CLDN9 proteins [11,13,14], we first analyzed the functionality of CLDN6/R209Q in these cells. Lentivectors expressing CLDN6/wt or CLDN6/R209Q were generated and used to transduce 293T cells. Both proteins were equally cell surface expressed, as controlled by flow cytometry (Fig 2A) and immunofluorescence (Fig 2B). Next, transduced 293T cells were infected with HCVpp1a and HCVpp2a expressing the *Firefly*-Luciferase reporter gene. However, no significant luciferase activity was observed in cells expressing either CLDN6/wt or CLDN6/R209Q (Fig 2C). Since it has been shown that permissiveness of 293T cells can be optimized by overexpression of HCV entry factors [49], we overexpressed SRB1, CD81, OCLN alone or in combination in 293T-CLDN6 cells. As shown in Fig 2D, most of the combinations did not lead to an increase of cell permissiveness. In contrast, we managed to increase HCVpp entry when 293T-CLDN6 cells were complemented with SRB1, CD81 and OCLN (Fig 2E). However, we did not observe any significant effect of the R209Q mutation on the functionality of CLDN6 in HCVpp entry (Fig 2E).

**Fig 3:**
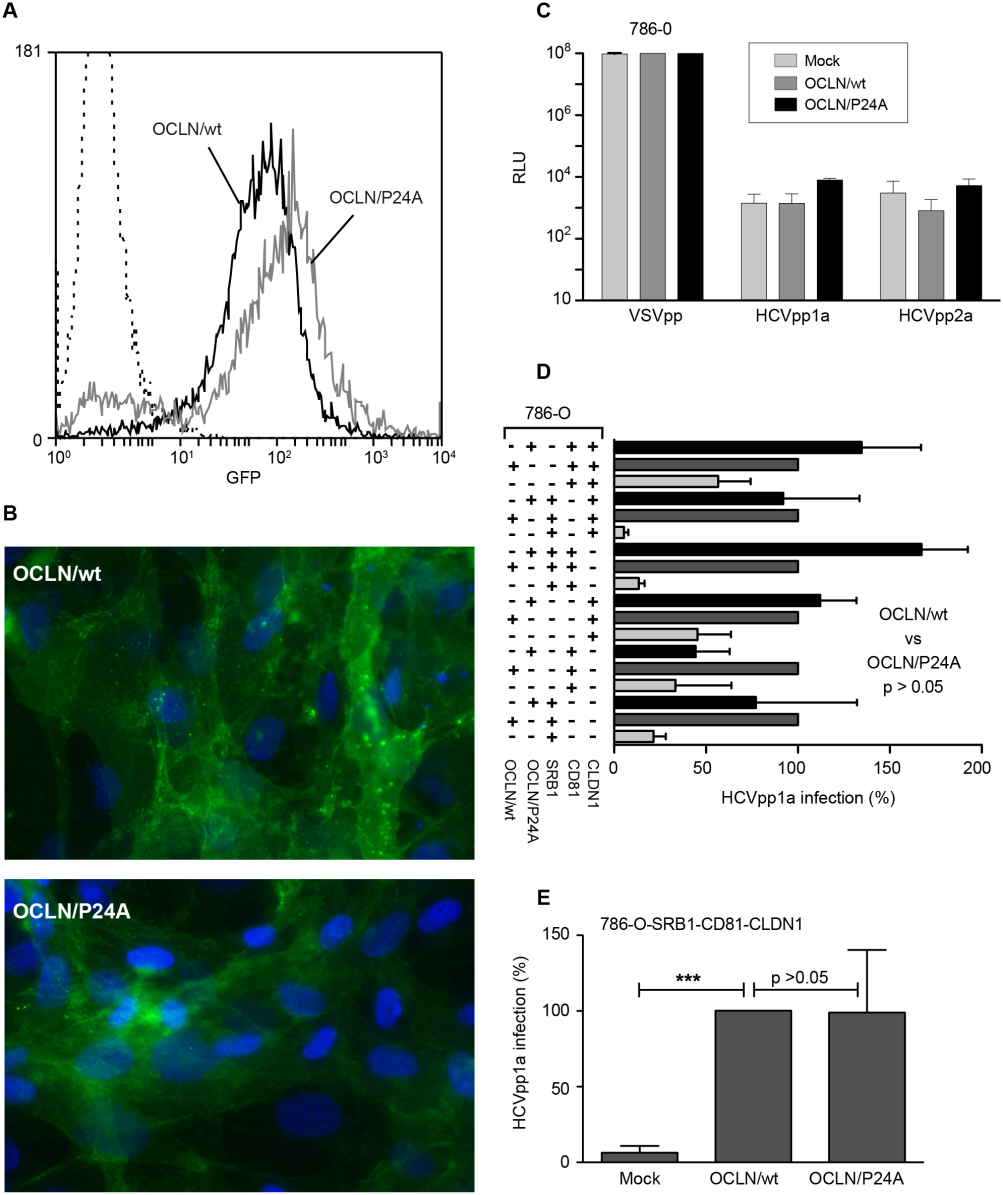
OCLN/P24A mutation does not affect HCVpp entry. 786-O cells were transduced twice at 24h interval with OCLN/wt or OCLN/P24A. Expression was analysed 48h after the last transduction round. (A) OCLN/wt and OCLN/P24A expression in 786-O cells was estimated through the percentage of GFP positive cells by flow cytometry analysis. (B) OCLN/wt and OCLN/P24A export at the cell surface in transduced 786-O cells was analysed by microscopy. (**C**) Two days after the last transduction, 786-O cells were infected with HCVpp1a, HCVpp2a or VSVpp expressing the *Firefly*-luciferase reporter gene. Non-transduced 786-O cells (Mock) were infected in parallel. Results are presented in relative luciferase units (RLU). (**D**) 786-O cells were transduced sequentially with a combination of lentivectors encoding SRB1, CD81 and CLDN1 in addition to OCLN/wt or OCLN/P24A. Cells were infected 48h after the last transduction round with HCVpp1a or VSVpp. Cells were lysed 72h post-infection and results were normalised with luciferase activities measured in cells infected with VSVpp. Values were adjusted to 100% infection for cells expressing OCLN/wt. (**E**) 786-O cells transduced to express SRB1, CD81 and CLDN1 in addition to OCLN/wt or OCLN/P24A were infected with HCVpp1a or VSVpp. Cells were lysed 72h post-infection and results were normalised with luciferase activities measured in cells infected with VSVpp. Values were adjusted to 100% infection for cells expressing OCLN/wt. Results are presented as mean ± SD of four independent experiments. *** means a *p* value below 0.001.

With the same approach as in 293T cells, we used the 786-O cell line, which expresses very low levels of OCLN [11], to study the effect of the P24A mutation on the functionality of OCLN in HCV entry. We transduced 786-O cells with lentivectors expressing OCLN/wt and OCLN/P24A fused to GFP. Both proteins were equally expressed, as controlled by flow cytometry (Fig 3A) and immunofluorescence (Fig 3B). As in 293T cells, 786-O cells expressing OCLN proteins were not permissive to HCVpp infection (Fig 3C). We therefore optimized cell permissiveness by overexpressing other entry factors. After infection with HCVpp, satisfying infection levels were obtained when cells expressed at least SRB1 in combination with OCLN/wt or OCLN/P24A (Fig 3D and 3E). However, for all combinations tested, no significant difference was observed between cells expressing OCLN/wt or OCLN/P24A (Fig 3D and 3E). On the whole, our results indicate that OCLN/P24A supports HCVpp entry in 786-O cells.

We also analysed the functionality of CLDN6/R209Q and OCLN/P24A expressed alone or in combination in TZM cells, in which coexpression of CLDN1 and OCLN allows HCVpp entry [11]. In accordance with our previous observations, HCVpp infection of transduced TZM cells showed no effect of mutations on the functionality of CLDN-6 and OCLN in HCV entry (data not shown).

### Search for a dominant negative effect of CLDN6 and OCLN variants expressed in hepatic cell lines

Since we observed no effect of the two mutations expressed separately in our previous systems, we hypothesized that a dominant negative effect in a more relevant cell system could explain the possible resistance of the patient to HCV in which the mutations were found. Indeed, the patient is a compound heterozygous for both mutations. Therefore, we overexpressed wt or mutated forms of CLDN6 and OCLN in hepatoma Huh-7 cells that endogenously express CLDN1, -6 and OCLN proteins.

**Fig 4:**
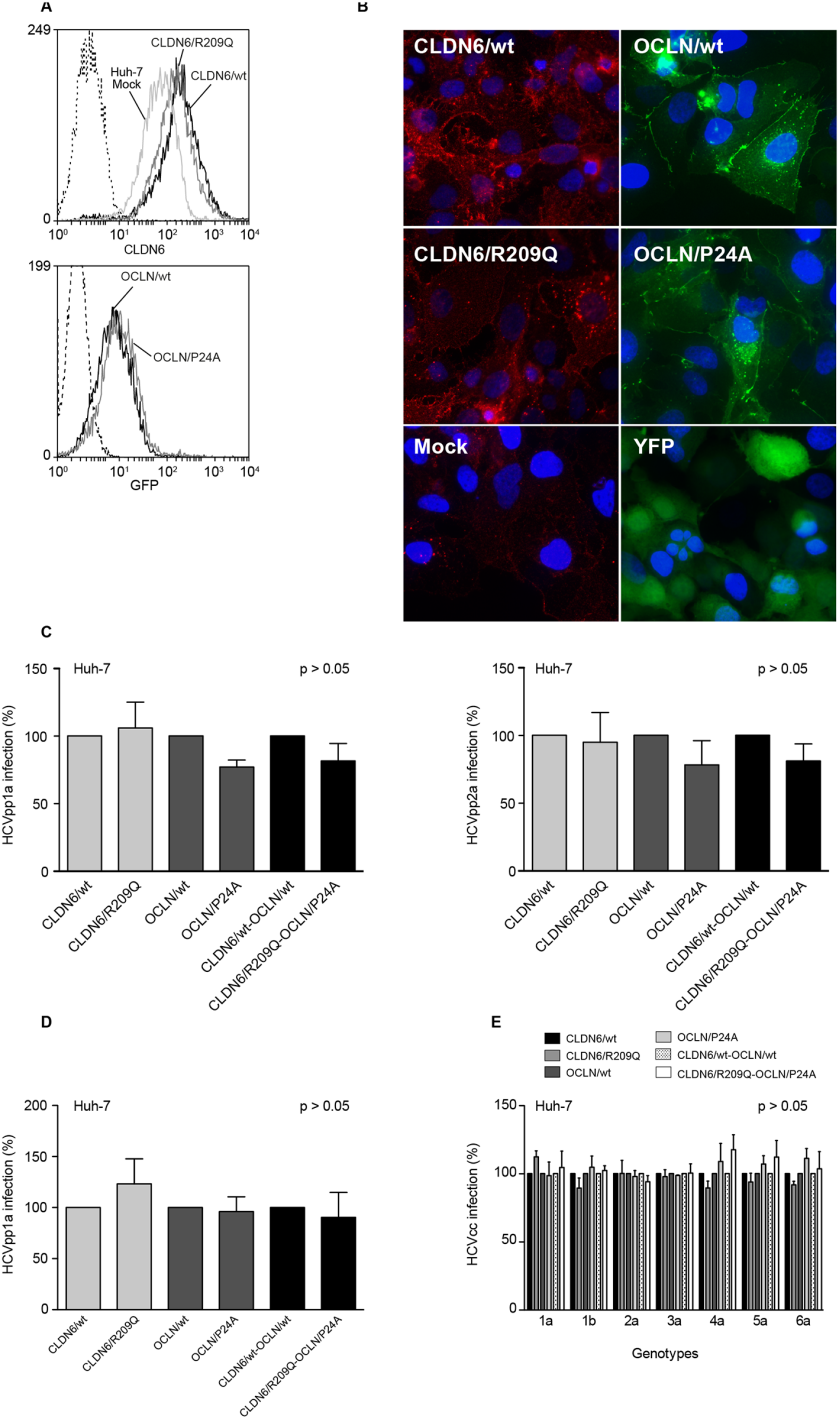
Expression of CLDN6/R209Q and OCLN/P24A mutants in Huh-7 cells and analysis of their functionality in HCV infection. Huh-7 cells were sequentially transduced twice at 24h interval with lentivectors expressing CLDN6/wt or CLDN6/R209Q and/or OCLN/wt or OCLN/P24A. Two days after the last transduction round, protein expression was controlled and cells were infected. (**A**) Expression in transduced Huh-7 cells. Upper panel, CLDN6/wt and CLDN6/R209Q cell surface expression was measured by flow cytometry using an anti-CLDN6 mAb, and compared to that of non-transduced Huh-7 cells (Huh-7 Mock). Cells stained with secondary antibodies were used as negative controls (dashed line). Lower panel, GFP-tagged OCLN/wt and OCLN/P24A expression in Huh-7 cells was estimated through the percentage of GFP positive cells by flow cytometry analysis. Huh-7 Mock were used as negative controls (dashed line). (**B**) CLDN6/wt and CLDN6/R209Q expression in Huh-7 cells was verified by indirect immunofluorescence using an anti-CLDN6 mAb and compared to non-transduced Huh-7 cells (Mock). Cell surface expression of GFP-tagged OCLN/wt and OCLN/P24A in Huh-7 cells was determined by fluorescent microscopy. Huh-7 cells transduced with a lentiviral vector expressing the Yellow Fluorescent Protein (YFP) were used as positive controls. Transduced Huh-7 cells were infected with HCVpp1a, HCVpp2a or VSVpp, expressing the *Firefly*-luciferase reporter gene (**C**), or HCVcc2a expressing the *Gaussia*-luciferase reporter gene (**D**). HCVpp values were normalized with luciferase activities measured in cells infected with control VSVpp. HCVpp and HCVcc values were adjusted to 100% infection for cells expressing the wt forms of CLDN6 and OCLN. (**E)** Transduced Huh-7 cells were infected with JFH1-based chimeras expressing structural proteins from genotypes 1 to 6, as indicated. Infection levels were quantified by indirect immunofluorescence. Values were adjusted to 100% infection for cells expressing the wt forms of CLDN6 and OCLN. Results are presented as mean ± SD of at least three independent experiments.

After controlling cell surface expression (Fig 4A and 4B), cells were infected with HCVpp1a or HCVpp2a and luciferase activities were measured 72h post-infection. Results were normalised with cells infected with VSVpp. HCVpp1a and HCVpp2a infections showed no significant difference in permissiveness of Huh-7 cells expressing CLDN6/wt or CLDN6/R209Q, OCLN/wt or OCLN/P24A or a combination of both proteins (Fig 4C).

**Fig 5:**
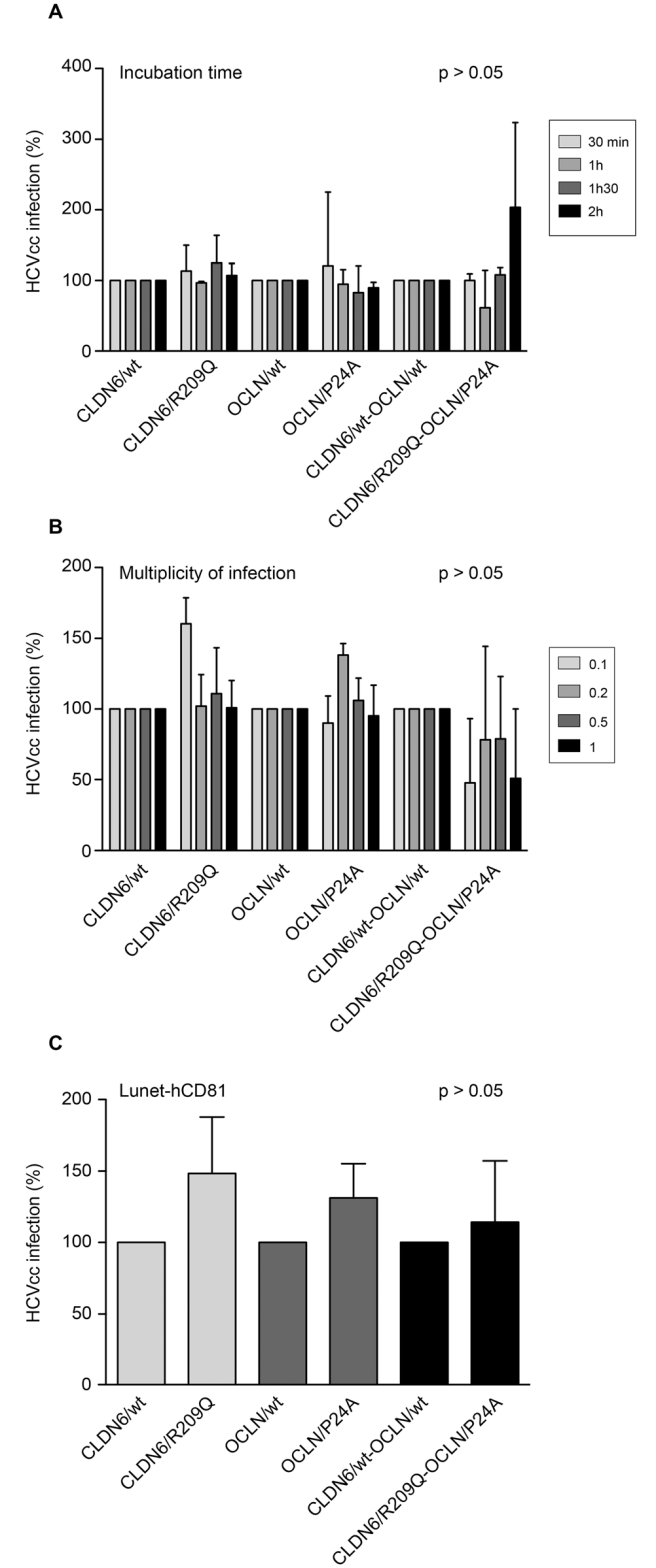
CLDN6/R209Q and OCLN/P24A mutations do not affect the kinetics of HCV entry and cellular physiology. Huh-7 cells were transduced with CLDN6/wt or CLDN6/R209Q and OCLN/wt or OCLN/P24A either alone or in combination. (**A**) Forty-eight hours after the last transduction round, cells were infected with HCVcc for 2h, 1h30, 1h or 30min. After infection, cells were rinsed and fresh medium was added. At 30h post-infection, cells were lysed and *Gaussia*-luciferase activities were measured. Results were normalized to luciferase activities measured in cells expressing the wt forms of CLDN6 and OCLN. (**B**) Cells were infected with HCVcc at indicated m.o.i. in serum-free DMEM for 2 h. At 30 h post-infection, cells were lysed and *Gaussia*-luciferase activities were measured. Results were normalized to luciferase activities measured in cells expressing the wt forms of CLDN6 and OCLN. Results are presented as mean ± SD of two independent experiments. (**C**) Huh-7-Lunet-CD81-FLuc cells, which endogenously expressed the *Firefly*-luciferase reporter gene, were transduced with CLDN6/wt or CLDN6/R209Q and OCLN/wt or OCLN/P24A either alone or in combination. Forty-eight hours after the last transduction round, cells were infected with HCVcc expressing the *Gaussia*-luciferase reporter gene. At 30h post-infection, cells were lysed and luciferase activities were measured with the Dual-luciferase^®^ reporter assay system (Promega). *Gaussia*-luciferase activities were normalized to Huh-7-Lunet-CD81 cells *Firefly*-luciferase activities. Results were adjusted to 100% infection for cells expressing the wt forms of CLDN6 and OCLN. Results are presented as mean ± SD of at least three independent experiments.

Next, we infected transduced Huh-7 cells with HCVcc (JFH-1, genotype 2a) expressing a *Gaussia*-luciferase reporter gene. Luciferase activities were measured 30h post-infection. As shown in Fig 4D, permissiveness of Huh-7 cells was not affected by the CLDN6 and OCLN mutations. Since a differential CLDN and OCLN usage by HCV isolates has been described [50,51], we next infected transduced Huh-7 with a series of JFH1-based chimeras expressing structural proteins from genotypes 1 to 6 [33]. Infection levels were quantified by indirect immunofluorescence (Fig 4E). Expression of mutated CLDN6 and/or OCLN did not affect permissiveness of Huh-7 cells to all HCV genotypes. Long-term expression of mutants (3 weeks) in Huh-7 cells before infection did not affect either cell permissiveness to HCVpp and HCVcc (data not shown). Moreover, the change of incubation time or multiplicity of infection (m.o.i.) during viral inoculation of Huh-7 cells in order to detect an effect of the mutations on kinetics of adsorption or on entry efficiency, respectively, did not show any effect of mutants on HCVcc infection levels (Fig 5A and 5B).

In order to exclude a potential effect of CLDN6 and OCLN variants on cellular physiology, we also expressed them in Huh-7-Lunet-hCD81-FLuc cells (data not shown), which endogenously express the *Firefly*-luciferase (FLuc) reporter gene [25]. Lunet cells were infected with HCVcc and values normalized with FLuc activities. As shown in Fig 5C, single or combined expression of CLDN6/R209Q and OCLN/P24A mutants did not affect the permissiveness of Lunet cells to HCV infection, confirming our previous results.

It has to be noted that additional experiments showed that mutations have also no effect on HCV replication and assembly (data not shown).

Taken together, our results show that the identified mutations do not have any dominant negative effect on HCV infection in widely used hepatoma cell models.

### Effect of CLDN6 and OCLN variants on cell-to-cell transmission

**Fig 6:**
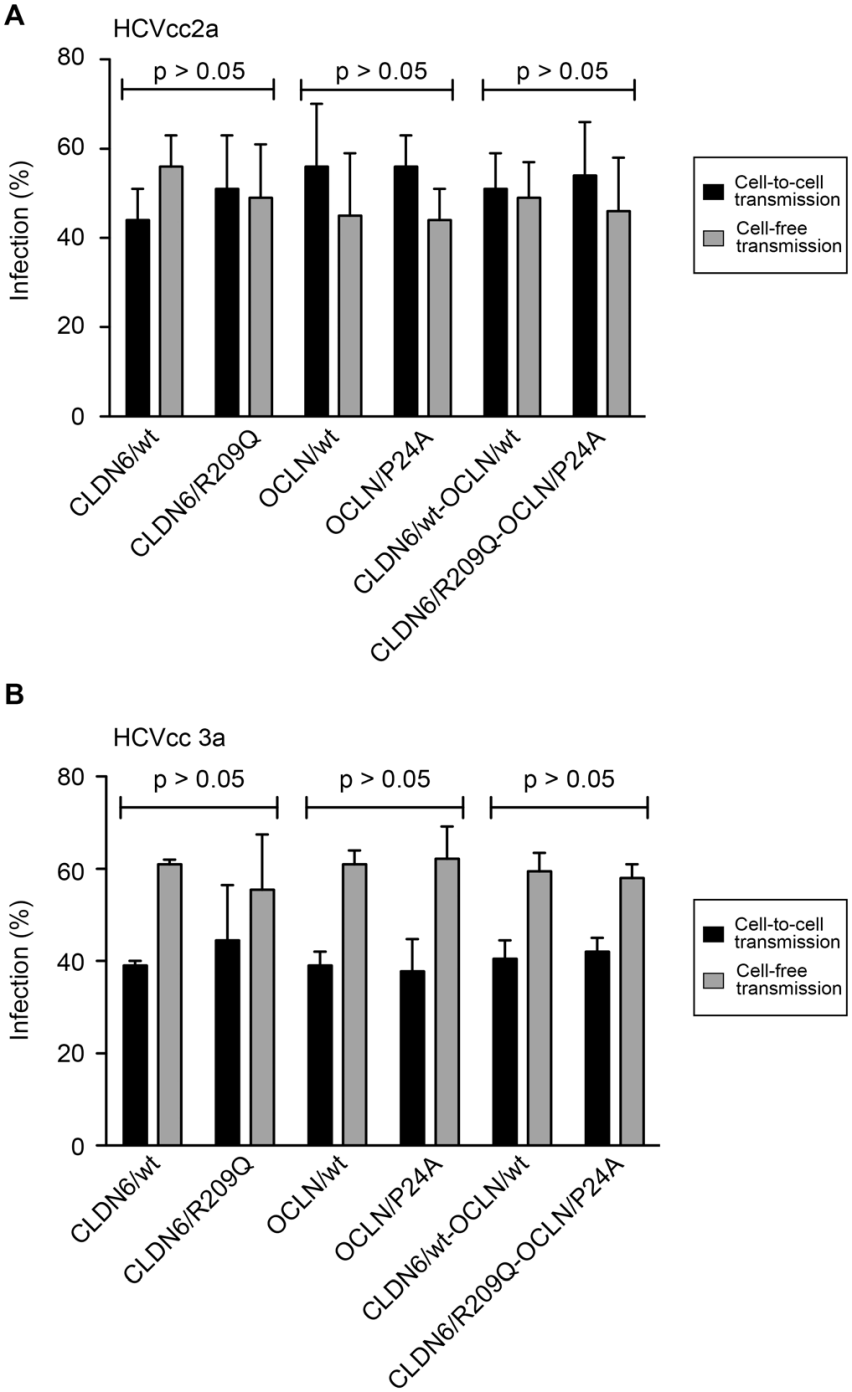
CLDN6/R209Q and OCLN/P24A mutations do not affect HCV cell-to-cell transmission. Huh-7 cells were transduced with CLDN6/wt or CLDN6/R209Q mutant and OCLN/wt or OCLN/P24A mutant either alone or in combination. Transduced cells were cocultivated for 24 h with CMFDA-stained donor cells infected with (**A**) HCVcc 2a or (**B**) HCVcc 3a in the presence (cell-to-cell transmission) or in the absence (cell-free and cell-to-cell transmission) of the 3–11 neutralizing antibody, which prevents the cell-free transmission. Cells were stained with an anti-NS5 antibody and analyzed by flow cytometry. HCV-positive CMFDA-negative cell populations were considered as newly infected cells through cell-to-cell transmission. Results of cell-free (light grey) and cell-to-cell (black) transmission are presented as percentages relative to the total transmission.

HCV entry can occur either through newly secreted virions or directly through transmission from one cell to another. The mechanism of HCV cell-to-cell transmission is mostly unknown, but CLDN1 and OCLN seem to be involved in this process [52–55]. Even if our first results showed that the two mutations did not impact HCV entry, it cannot be excluded that the mutations impact cell-to-cell transmission and that the lack of effect on cell-free entry might hide an effect on cell-to-cell transmission. We therefore analyzed the effect of mutations in CLDN6 and OCLN on cell-to-cell transmission, as described previously [56]. Briefly, Huh-7 cells were infected with HCVcc (genotype 2a or 3a) and then labeled with 5-chloromethylfluorescein diacetate (CMFDA). These donor cells were next cocultivated with naïve Huh-7 cells expressing wt or mutated forms of CLDN6 and OCLN, in the presence (cell-to-cell transmission) or in the absence (cell-free and cell-to-cell transmission) of anti-E2 neutralizing antibody (3–11), which prevents the cell-free transmission. After 24h, newly infected cells were quantified by flow cytometry, and HCV-positive CMFDA-negative cell populations were considered as newly infected cells through cell-to-cell transmission. As expected, cell-free transmission was not significantly different in cells expressing the mutated forms of CLDN6 or OCLN (Fig 6A and 6B). The analysis of cell-to-cell transmission showed that this transmission route was also not affected by the mutations in CLDN6 or OCLN, whatever the genotype of HCV (Fig 6A and 6B).

Altogether our results demonstrate that the mutations in CLDN6 and OCLN do not affect cell-free entry nor cell-to-cell transmission of HCV.

### Effect of CLDN6 and OCLN variants expressed in polarized HepG2 cells and Primary Human Hepatocytes

**Fig 7:**
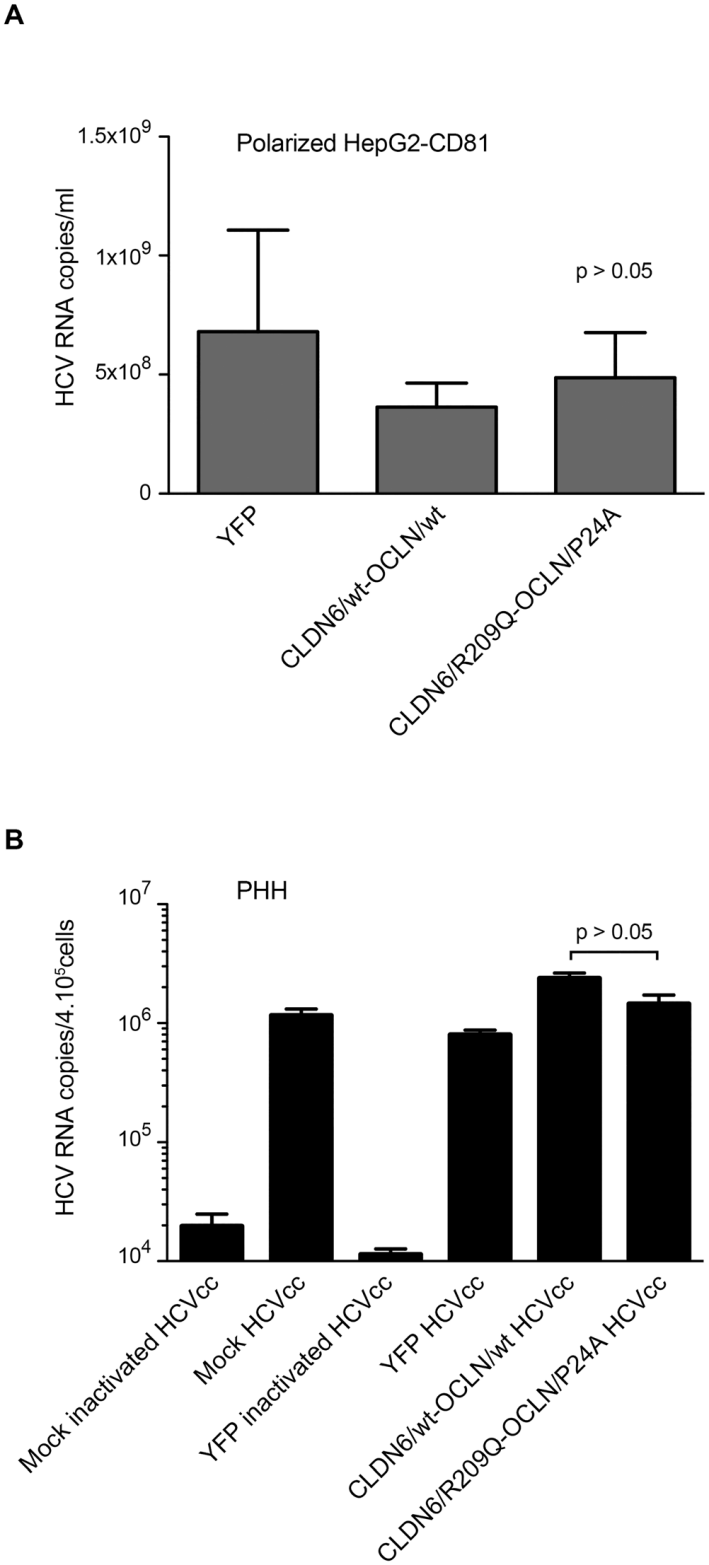
Functionality of CLDN6/R209Q and OCLN/P24A in HCV infection expressed in polarized cells or primary hepatocytes. (**A**) Two days after the last transduction round, HepG2-CD81-1SC3 cells were plated on transwells and polarization was induced. Once polarized, cells on the lower chamber of the transwell were infected with HCVcc2a. At 30h after infection, cells were lysed, total RNA was extracted and viral RNA was quantified by qRT-PCR. Results shown are from one experiment representative of three independent experiments. (**B**) Transduced PHH (Biopredic) were infected with cell culture adapted HCVcc2a [36]. Non-transduced PHH (Mock) and PHH transduced with a lentiviral vector expressing only the YFP were used as controls. Virus that had been inactivated at 60°C for 30 min (inactivated HCVcc) was used as another control. Infection levels were evaluated by quantifying HCV RNAs. Two independent experiments on PHH were performed. Results presented in B are the mean ± SD of triplicates done on PHH from one liver resection.

CLDN6 and OCLN are tight junction proteins, which are important in organization of polarized cells. Moreover, it has been shown that hepatocyte polarization plays an important role in regulation of HCV infection [57–59]. Therefore, we hypothesized that the effect of the mutations could only be observed in polarized cells. We transduced and infected a HepG2 cell clone that polarizes in the presence of DMSO (HepG2-CD81-ISC3 cells from S. Belouzard; unpublished data) [60]. Cells transduced with a lentiviral vector expressing only the Yellow Fluorescent Protein (YFP) were used as controls. CLDN6 and OCLN expression levels in transduced HepG2-CD81-ISC3 were controlled by flow cytometry and immunofluorescence (data not shown), as previously described. Two days after the last transduction round, cells were plated on transwells and when cells were confluent, polarization was induced by treating cells with DMSO. Polarization was controlled by immunostaining with an anti-ZO-1 antibody (data not shown). The cells were infected with HCVcc the day of their polarization and RNA from supernatants and cell lysates were extracted at 30h post-infection. Infection levels were quantified by qRT-PCR measurements of viral RNA. As shown in Fig 7A, there was no difference in the number of HCV RNA copies between cells expressing wt or mutated forms of CLDN6 and OCLN.

Together, these results demonstrate that the mutations in CLDN6 and OCLN have no effect on HCVcc infection in the context of polarized cells.

Finally, we assessed the effect of mutations in primary human hepatocytes (PHHs), the most physiologically relevant model to investigate HCV infection *in vitro*. Transduced PHHs expressing YFP, wt or mutated CLDN6 and OCLN proteins were infected with HCVcc or inactivated virus, as described previously [36]. Quantification of intracellular HCV RNAs showed that HCVcc efficiently infected PHHs compared to the inactivated virus. However, we did not observe any detectable effect of CLDN6/R209Q and OCLN/P24A expression on PHHs permissiveness (Fig 7B).

## Discussion

Only few patients clear HCV spontaneously and up to 80% of infected people will develop a chronic infection. Spontaneous clearance of infection can be predicted by some factors, such as the limited evolution of viral quasispecies, gender, HCV genotype, immune system specificities, as well as a symptomatic or asymptomatic acute infection [61–63]. In this way, polymorphisms within the interferon lambda gene region are predictive factors for both spontaneous HCV clearance and response to pegylated interferon/ribavirin therapy [64–68], [69]. Moreover, as several polymorphic variations in the gene coding the CCR5 coreceptor that abolish HIV infection have been described [70,71], it cannot be excluded that polymorphisms in HCV cell factors could yield resistance to HCV infection. We previously conducted a case-control study involving a cohort of HIV-infected IVDU at high risk of HCV infection but not infected, and identified in the same HIV+ HCV- patient one mutation in CLDN6 with no allele frequency described in the databases and one rare variant in OCLN [24]. In the present study, we conducted functional *in vitro* studies of these CLDN6 and OCLN variants to define whether they could be involved in resistance mechanisms. We analyzed the functionality of CLDN6/R209Q and OCLN/P24A in HCV entry. The use of different cellular models and HCV genotypes showed that variants of CLDN6 and OCLN supported cell entry by HCVpp and HCVcc with an efficiency similar to wildtype entry factors, as previously observed for CD81 and OCLN variants [53,72]. However, *in vitro* systems do not necessarily reflect *in vivo* events in the patient since interaction with other factors is predicted to occur *in vivo*. The use of humanized mice might be a more valuable model to study the functional impact of these mutations *in vivo*. Indeed, we cannot exclude that these variants found in the same patient might confer resistance to HCV infection *in vivo*. HCV entry is a complex and multistep process, variants in other entry factors might also be necessary in combination with those we have identified, to affect HCV entry. Indeed, HCV resistance is likely multigenic.

In our cohort of HIV+ HCV- IVDU patients, HCV resistance does not involve frequent mutations in HCV entry factors [24] and the two natural variants characterized in the present study did not show any negative dominance on HCV entry. These findings indicate that host resistance to HCV may involve other mechanisms beside entry factor mutations, as suggested for HIV resistance. Indeed, a large proportion of individuals who remain HIV-uninfected despite repeated sexual exposure harbor no mutation in HIV entry co-receptors (reviewed in [70]).

In conclusion, our study highlights the complexity of HCV resistance mechanisms. Thus, whole genome studies would be promising to identify the different cellular factors implicated in HCV resistance, new factors still remaining to be identified.
